# Inorganic Colloidal Electrolyte for Highly Robust Zinc-Ion Batteries

**DOI:** 10.1007/s40820-021-00595-6

**Published:** 2021-02-11

**Authors:** Jiawei Gao, Xuesong Xie, Shuquan Liang, Bingan Lu, Jiang Zhou

**Affiliations:** 1 School of Materials Science and Engineering, Central South University, Changsha, 410083 People’s Republic of China; 2 School of Physics and Electronics, Hunan University, Changsha, 410082 People’s Republic of China; 3 Key Laboratory of Electronic Packaging and Advanced Functional Materials of Hunan Province, Central South University, Changsha, 410083 People’s Republic of China

**Keywords:** Zn-ion battery, Palygorskite, Inorganic, Colloidal electrolyte, Cycle stability

## Abstract

Zinc-ion batteries (ZIBs) is a promising electrical energy storage candidate due to its eco-friendliness, low cost, and intrinsic safety, but on the cathode the element dissolution and the formation of irreversible products, and on the anode the growth of dendrite as well as irreversible products hinder its practical application. Herein, we propose a new type of the inorganic highly concentrated colloidal electrolytes (HCCE) for ZIBs promoting simultaneous robust protection of both cathode/anode leading to an effective suppression of element dissolution, dendrite, and irreversible products growth. The new HCCE has high Zn^2+^ ion transference number (0.64) endowed by the limitation of SO_4_^2−^, the competitive ion conductivity (1.1 × 10^–2^ S cm^−1^) and Zn^2+^ ion diffusion enabled by the uniform pore distribution (3.6 nm) and the limited free water. The Zn/HCCE/α-MnO_2_ cells exhibit high durability under both high and low current densities, which is almost 100% capacity retention at 200 mA g^−1^ after 400 cycles (290 mAh g^−1^) and 89% capacity retention under 500 mA g^−1^ after 1000 cycles (212 mAh g^−1^). Considering material sustainability and batteries’ high performances, the colloidal electrolyte may provide a feasible substitute beyond the liquid and all-solid-state electrolyte of ZIBs.

**Supplementary Information:**

The online version contains supplementary material available at (10.1007/s40820-021-00595-6).

## Highlights

- The Zn/MnO_2_ cell with inorganic colloidal electrolyte demonstrates unprecedented durability over 1000 cycles.
- For the cathode, the presence of the protective film can inhibit the dissolution of manganese element and the formation of irreversible by-products.
- For the anode, it can reduce the corrosion and de-solvation energy, inhibit the growth of dendrite and irreversible by-products.

**Supplementary Information:**

The online version contains supplementary material available at (10.1007/s40820-021-00595-6).

## Graphical abstract

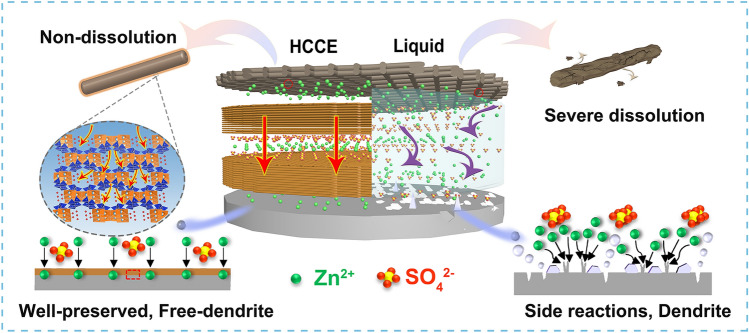

## Introduction

The high energy density, low cost, and the environmentally friendly nature of aqueous zinc-ion batteries (ZIBs) are attractive especially for the large-scale stationary electrical energy storage [1, 2]. Unfortunately, ZIBs suffer from the growth of dendrite [3], element dissolution [4], and the formation of irreversible products [5]. In order to solve these issues, great efforts have been devoted to the study on the new Zn-host cathode discovery (manganese-based oxides [6], vanadium-based oxides [7, 8], prussian blue analogous [9], and other organic materials [2] etc.), mechanism exploration in oxide/non-oxide material, aqueous/non-aqueous electrolyte [10, 11]. Moreover, much effort has been dedicated to the development of advanced metal Zn electrodes, from surface modification and structure designation (3D) to the alloy enhancement [12, 13]. However, these methods can hardly meet all the requirements. As a result, the cycling life and Coulombic efficiency (CE) of ZIBs are still far behind the state-of-the-art lithium-ion batteries.

It is well known, as a vital component of the ZIBs, the electrolyte provides the basic operating environment to guarantee the long-standing stability and endurability of the battery [14–16]. A high-quality electrolyte could improve the performance of ZIBs. For example, Wang et al. [17] and Zhao et al. [18] recently reported that “water-in-salt” or “water-in-deep eutectic solvent” [19] high-concentration electrolyte could exhibit a nearly 100% CE and result in dendrite-free Zn plating/stripping during operation. This concept makes use of the fact that the nature of the coordination environment of the Zn^2+^ cation in the solution can be changed on a molecular level, leading a completely different electrochemistry [20]. However, under extreme concentration conditions, it is hard to apply in large-scale because of high-cost organic and salt precipitation issues at low temperatures [21]. Therefore, major stride should be taken in electrolyte exploration.

Here, we propose a new inorganic high-concentration colloidal electrolyte (HCCE) induced by the palygorskite nano-inorganic material to replace the normal liquid electrolyte in an aqueous ZIBs. The palygorskite has an intermediate structure between the chain structure and the lamellar structure, and it belongs to the 2:1 layer-chain microstructure, where the lattice displacement and good adsorption of zinc ions that are expected to reduce the much stronger and tight solvation sheath with water. Based on the HCCE, the energy barrier of de-solvation for hydrated Zn^2+^ at the surface is only 32.3 kJ mol^−1^, which is almost 1.7 times barrier less than that of routine liquid electrolyte (52.5 kJ mol^−1^), suggesting the high electrolyte–electrode kinetics enabled by HCCE. As a result, The HCCE not only has the same order of ionic conductivity as liquid electrolyte, but also has a higher Zn^2+^ transference number. The HCCE could promote simultaneously building of a protective layer on the surface of both anode and cathode, consequently suppress Mn dissolution and lead to a well-preserved and free-dendrite metal Zn anode. The battery with HCCE achieves high Coulombic efficiency and longer cycle life, exhibiting excellent durability up to 400 cycles at 200 mA g^−1^ with no capacity fading (290 mAh g^−1^) and maintaining a specific capacity of 212 mAh g^−1^ after 1000 cycles at 500 mA g^−1^. It can be concluded that the HCCE provides a comprehensive enhancement, and offer an available and affordable avenue for the highly efficient utilization of Zn in Zn-based batteries.

## Experimental Section

### Material Synthesis

Preparation of *α*-MnO_2_: In a modified synthesis of *α*-MnO_2_ [22], 0.00225 mol MnSO_4_·H_2_O was added to 15 mL deionized water and stirred it until a clear solution was obtained. Then, 15 mL 0.1 M KMnO_4_ aqueous solution was slowly added into the above solution. The mixture was stirred at room temperature for 1 h. The solution was then transferred to a Teflon-lined autoclave and heated at 160 ℃ for 12 h. After cooling down, the obtained material was collected by centrifugation, washed three times with deionized water, and dried in an air oven at 60 ℃.

Preparation of a high-concentration colloidal electrolyte: 2 M ZnSO_4_ + 0.1 M MnSO_4_ was used as a liquid electrolyte. The high-concentration colloidal electrolyte (HCCE) was prepared by mixing palygorskite (800 mesh, Weifang Purun Trading Co. Ltd, China) and liquid electrolyte in a mass ratio of 3:10 (1:10, 2:10, and 4:10) which signed 30% (10%, 20%, and 40%). Before using, we will mix the raw palygorskite with a large amount of deionized water to form slurry evenly, centrifuge it at 8000 RPM for 5 min, and then remove the impurity. The HCCE (slurry) could be obtained by pouring palygorskite into the liquid electrolyte, mixing, stirring, and then under ultrasonic at least 60 min (it can be used after standing for 12 h without any other treatment).

### Material Characterization

X-ray diffraction (XRD) measurements were performed using a Rigaku D/max2500 powder diffractometer (Cu Kα, *λ* = 0.15405 nm). Determination of elements in solution by inductively coupled plasma optical emission spectrometry (ICP-OES). XPS spectra were performed on an ESCALAB 250Xi X-ray photoelectron spectrometer (Thermo Fisher) and C 1 s peak is 284.8 eV. SEM images were collected on a Nova Nano SEM 230 operating at 10 kV. The element analysis and morphology measurements were obtained by energy-dispersive X-ray (EDS) and transmission electron microscope (TEM, HEM-2100F/UHR). FTIR was performed with a Nicolet 6700 spectrometer instrument. The surface morphology of Zn anode was observed by AFM (CSPM 5000).

### Electrochemical Characterization

The cathode electrodes were prepared by coating a slurry of 70% *α*-MnO_2_, 20% acetylene black, and 10% polyvinylidene fluoride (PVDF) onto stainless steel wire mesh (SSWM) and then let it dry at 80 ℃ in vacuum overnight. Zn/MnO_2_ batteries were assembled using a glass fiber filter as a separator (Whatman GF/A), HCCE as the electrolyte and Zn foil as the anode (the HCCE was coated on both sides of separator) in CR2016 coin cells. The charge/discharge experiments were carried out on the multichannel battery testing system (LAND CT2001A). The CV and EIS tests were recorded using an electrochemical workstation (CHI660C). Galvanostatic intermittence titration technique (GITT) was measured by the Arbin test system.

## Result and Discussion

### Materials Synthesis and Characterization

**Fig. 1:**
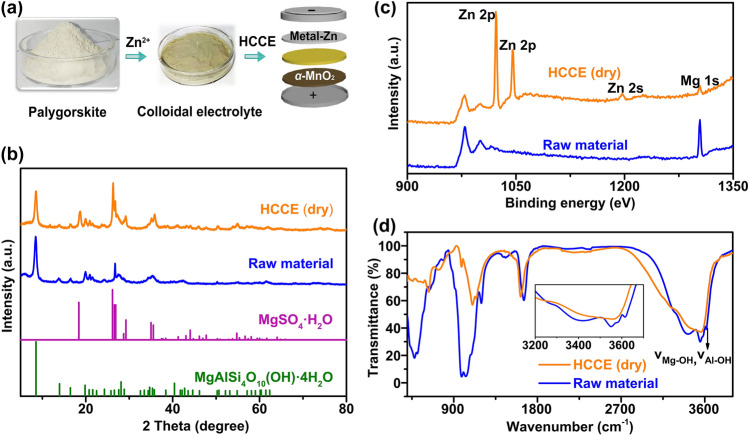
**a** A simple process of colloidal electrolyte preparation and application. **b** XRD patterns of raw material (palygorskite) and colloidal electrolyte (after drying). **c** XPS full-spectrum image of raw material (palygorskite) and colloidal electrolyte (after drying). **d** FTIR spectra of raw material (palygorskite) and colloidal electrolyte (after drying)

The inorganic colloidal electrolyte is prepared by directly added the normal liquid electrolyte (2 M ZnSO_4_ + 0.1 M MnSO_4_) to the raw palygorskite inorganic material, and the Tyndall effect is observed (Fig. S1). When the concentration of palygorskite is further increased, the white color material turns into yellow–brown and high viscosity (Figs. 1a and S2). Compared with the raw stick materials (Fig. S3), the palygorskite that immerses into liquid electrolyte illustrate a uniform distribution of Zn element around the stick in energy-dispersive X-ray (EDX) elemental mapping analyses (Fig. S4), confirming that Zn^2+^ did react with the palygorskite via absorption or ions exchange [23]. As shown in Fig. 1b, the XRD pattern of HCCE remains a characterized palygorskite phase composition like raw materials (JCPDS#29-0855), which confirmed the Zn or Mn ions did not change this phase structure merely by means of the isomorphic substitution reaction. While a new phase MgSO_4_·H_2_O (JCPDS#33-0882) appears in the colloidal electrolyte after drying, suggesting the replacement of lattice Mg by the Zn as the reported studies [23–25].

The XPS spectral characterization of the raw material (palygorskite) and the colloidal electrolyte is shown in Fig. 1c. For colloidal electrolyte powders, Zn 3*p* double peak (~ 1022.89 and 1045.97 eV) and Zn 2* s* peak (~ 1197.16 eV) are both observed and the intensity of Mg 1* s* peak (~ 1304.2 eV) is significantly reduced compared to that of raw ones, which proves that the large quantity of Mg in palygorskite is indeed replaced by Zn element. The Fourier transform infrared (FTIR) results show that the intensity of obvious absorption peak appearing at about 3615 cm^−1^ (υ_(Mg, Al)-OH_) and 3585 cm^−1^ (υ$_{{\text{Mg}}_{2}-{\text{OH}}}$) is largely reduced (Fig. 1d), which is well established in the literature as being because of υ_(Mg, Al)-OH_ and υ$_{{\text{Mg}}_{2}-{\text{OH}}}$ stretching mode of raw materials [26, 27]. This indicates that the strong interaction of Mg-OH may be replaced by the Zn-OH group. To conclude, the equation for the HCCE can be summarized as below [28, 29]:

1 $$ \begin{gathered}   {\text{MgAlSi}}_{4} {\text{O}}_{{10}} \left( {{\text{OH}}} \right) \cdot 4{\text{H}}_{2} {\text{O}} + y{\text{ Zn}}^{{2 + }}  \hfill \\    \leftrightarrow {\text{Zn}}_{y} {\text{Mg}}_{{1 - y}} {\text{AlSi}}_{4} {\text{O}}_{{10}} \left( {{\text{OH}}} \right) \cdot 4{\text{H}}_{2} {\text{O}} + y{\text{Mg}}^{{2 + }}  \hfill \\  \end{gathered}  $$

Based on the analysis, it indicates that Zn^2+^ strongly interacts with the palygorskite, enabling Zn^2+^ insertion among internal crystals (isomorphic substitution reaction), the Zn^2+^ is not exposed to the environment surrounded by solvent molecules (H_2_O) for a long time. Next, the effect of the HCCE based on the limited liquid water on the electrode and battery performance will be analyzed.

### Electrochemical Performance of Zn/MnO_2_ Cells Cycled with HCCE

**Fig. 2:**
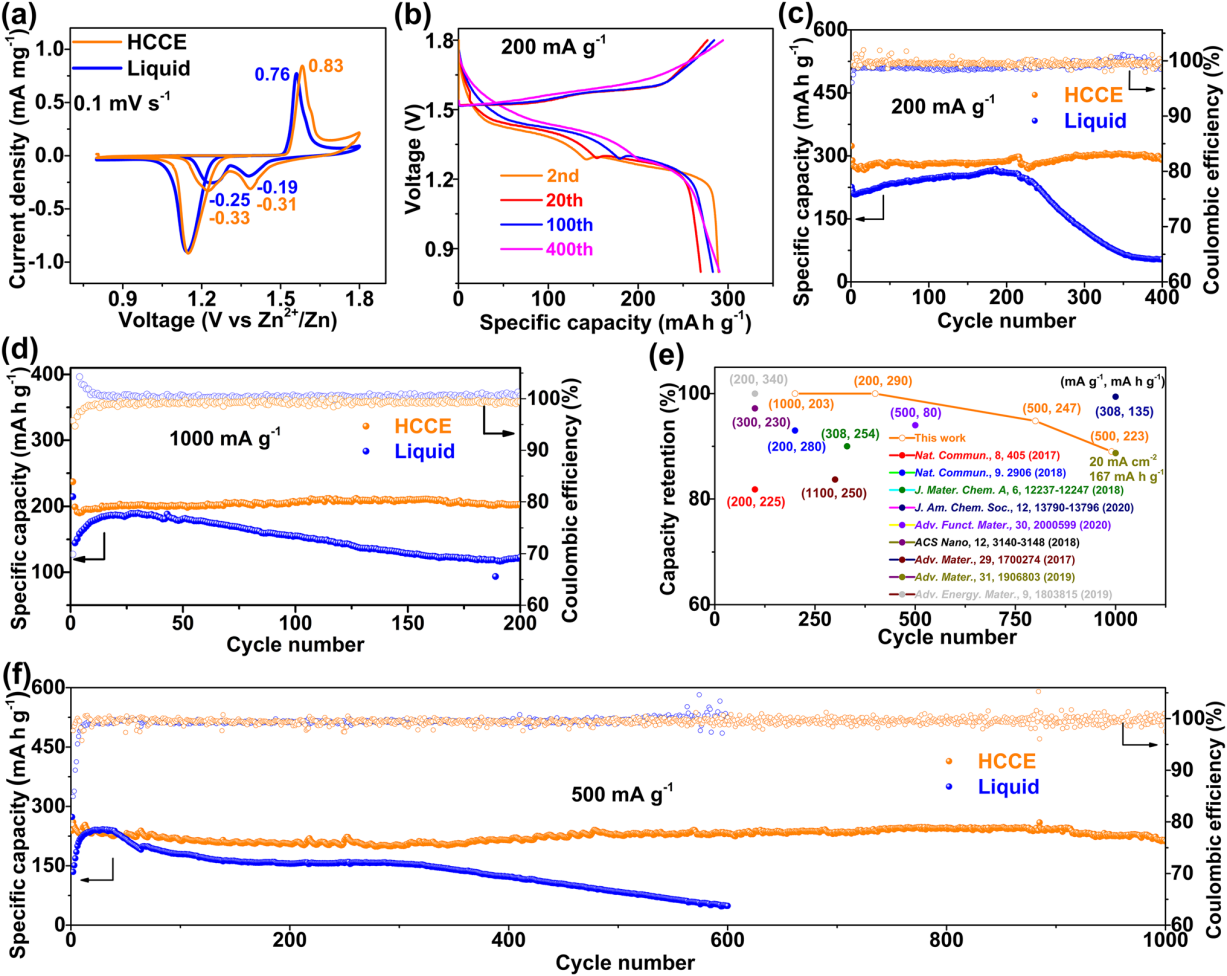
**a** CV curves (the first cycle) at a scan rate of 0.1 mV s^−1^ with the HCCE and liquid electrolyte. **b** Charge–discharge curves under different cycles with the HCCE at 200 mA g^−1^. **c** Cycling performance of the cell with HCCE and liquid electrolyte cells at 200 mA g^−1^. **d** Cycling performance of the cell with HCCE and liquid electrolyte cells at 1000 mA g^−1^. **e** Capacity retention versus cycle number for electrolyte reported in aqueous Zn/mangan-based materials (mainly manganese dioxide) batteries, note: the current density and final specific capacity are indicated in the figure. **f** Long-life cycling performance of the cell with HCCE and liquid electrolyte at 500 mA g^−1^

The cyclic voltammetry curves of HCCE showed it owns 0.83 mA mg^−1^ peak current at charge state and  0.33 mA mg^−1^,  0.31 mA mg^−1^ peak current at discharge state which is higher than that of liquid with 0.76 mA mg^−1^ charge peak current,  0.25 and  0.19 mA mg^−1^ discharge peak current at the scan of 0.1 mV s^−1^, which means that HCCE has the higher electrochemical reactivity and specific capacity than liquid as shown in Fig. 2a. As displayed in Fig. S5b, the cell with HCEE possesses considerably higher capacities than that of the cell with a liquid electrolyte at each rate in the voltage range of 0.8–1.8 V (*vs*. Zn^2+^/Zn). Figure 2b shows the charge/discharge curves of the cell with HCCE cycled at a current rate of 200 mA g^−1^. After the first cycle of activation, the cell with HCCE maintains stable capacities of around 290 mAh g^−1^ with a high CE of around 100% and well stable voltage-platform as well as high capacity retention (almost 100% after 400 cycles) under the low current density as shown in Fig. 2c (the comparison of circulating performance electrolyte with different palygorskite concentration is shown in Fig. S6). Even at high 1000 mA g^−1^ current density, the cell cycled with HCCE delivers a nearly 100% capacity retention after 200 cycles with a specific capacity of 202 mAh g^−1^ as shown in Fig. 2d. For the long-standing performance, the cell equipped with HCCE possessed superior durability over 1000 cycles at 500 mA g^−1^ (Fig. 2f) than that of the liquid electrolyte with same conditions. Specifically, the last ten charge/discharge curves of the cell at 1000 still remain the high operating voltage (about 1.35 V, see Fig. S7), further demonstrating the high stability and reversibility of the cell with HCCE, Accordingly, Fig. 2e shows the comparison of cycle number and capacity retention based on different electrolytes in mangan-based materials (mainly manganese dioxide) in recent years, the cell cycled with HCCE shows the superior electrochemical performance compared to others.

The EIS data of the Zn/MnO_2_ battery (Fig. S5c), fitted by the equivalent circuit shown in the inset of Fig. S5c, shows a decrease of charge transfer resistance with the increase of concentration of colloidal, which suggests the good conductivity and high electrolyte–electrode kinetics enabled by HCCE. Table S1 presents an ohmic internal resistance (*R*_s_, Liquid, 3.2 Ω; *R*_s_, Colloidal, 2.1 Ω) and a charge transfer resistance (*R*_ct_, Liquid, 1694 Ω; *R*_ct_, Colloidal, 297 Ω), respectively. Besides, the HCCE can effectively suppress the self-discharge phenomenon (Fig. S5d). The one of main contributions to the self-discharge is the faradaic reaction, which includes the decomposition of the electrolyte and the redox side reactions caused by impurities or functional groups on the electrode surface [30–32]. This indicates that the HCCE is capable of inhibiting side reactions during the practical application. In order to figure out the mechanism of performance enhancement for HCCE, the characterizations were conducted on the electrodes and electrolyte in the following part.

### Analysis of Cathode of Zn/MnO_2_ Battery

**Fig. 3:**
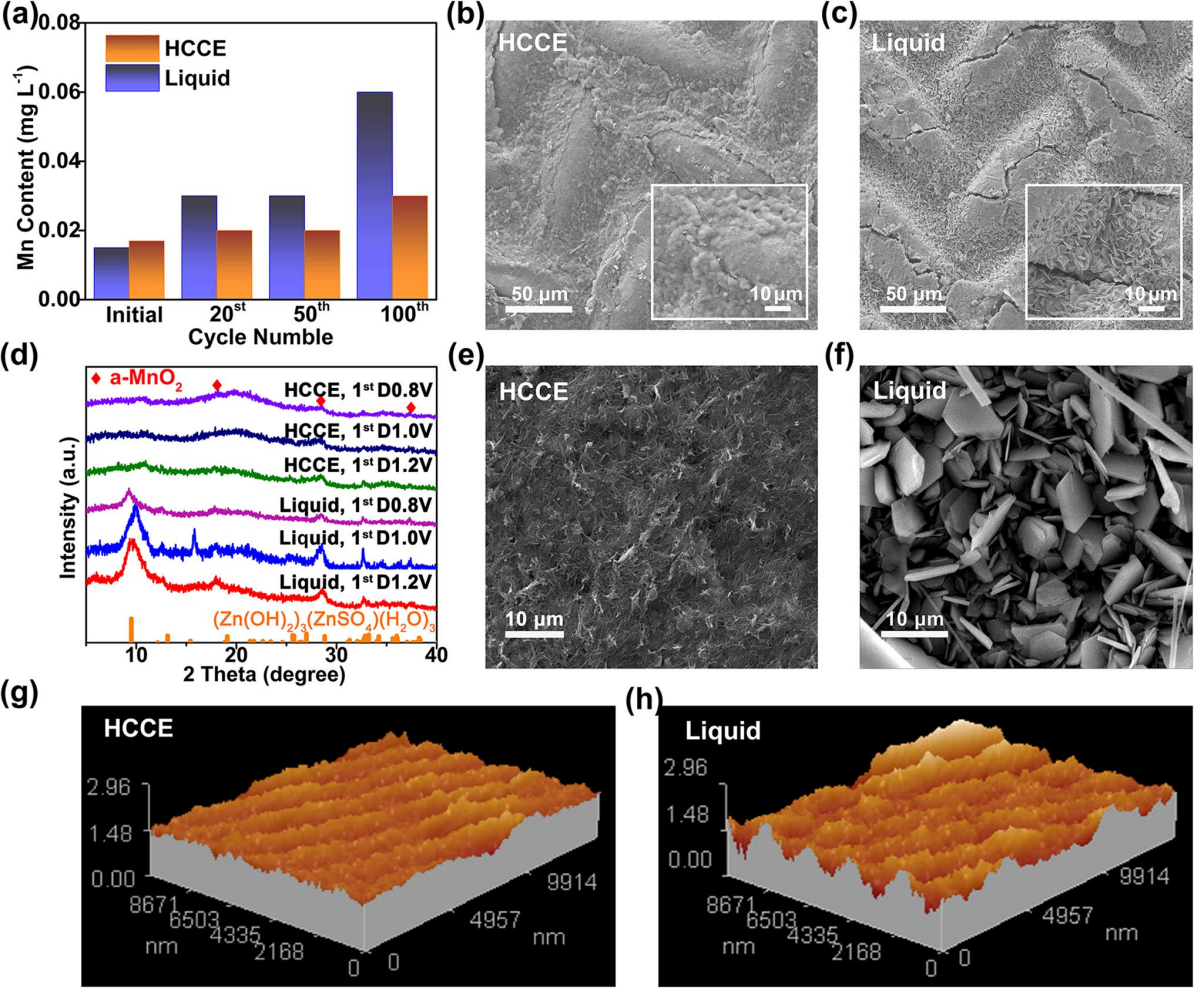
**a** Element analysis of dissolved Mn^2+^ in Zn/MnO_2_ battery during cycling with 2 M ZnSO_4_ aqueous electrolyte and colloidal electrolyte (2 M ZnSO_4_ + palygorskite). SEM images of **b** cathode of the battery with HCCE and **c** the battery with liquid electrolyte after initial fully discharge to 1.0 V at 200 mA g^−1^. **d** ex-situ XRD patterns of the cell with HCCE and liquid electrolyte discharged/charged to different voltage states at 200 mA g^−1^. SEM images of **e** anode of the battery with HCCE and **f** the battery with liquid electrolyte cycled for 200 cycles 1000 mA g^−1^. AFM images of **g** anode of the battery with HCCE and **h** the battery with liquid electrolyte cycled for 200 cycles 1000 mA g^−1^

The MnO_2_ is of interest due to its low cost, moderate discharge potential, and acceptable rate/cycle performance, as well as its high theoretical capacity of about 308 mAh g^−1^ (Zn_0.5_MnO_2_). However, It is well known that the dissolution of Mn is a major issue for Mn-based electrodes due to Jahn–Teller effect [33, 34], leading to capacity fading and short cycle life [35]. To analyze the superior performance, the inductively coupled plasma optical emission spectrometry (ICP-OES) was conducted to evaluate Mn concentration in Zn/MnO_2_ battery during cycling. It shows that the dissolution of the Mn element could be effectively alleviated by the colloidal electrolyte (with 2 M ZnSO_4_ in the absence of MnSO_4_) at each cycle (Fig. 3a). For the postmortem electrode a well-preserved cathode morphology is achieved after discharging to 1.0 V at 200 mA g^−1^ (Fig. 3b), 200 cycles at 1 A g^−1^ (Fig. S8), and 400 cycles at 0.2 A g^−1^ (Fig. S9), respectively, whereas the liquid one delivers a clearly dissolved feature compared to HCCE (Fig. 3c), which may be caused by the severe Mn dissolution and exacerbated by the acute acid due to local pH fluctuation as the previous study reported [36]. To further analyze, the ex-situ Transmission electron microscopy (TEM) test of cathode discharged to 0.8 V after being cycled with HCCE and liquid was performed. It was found that the diameter of MnO_2_ nanorod after being cycled with liquid was only 8.1 nm (Fig. S10), whereas the HCCE one keeps around 43 nm and is surrounded by a layer of membrane (the fiber shapes) as result of the continuous Mg and Zn but not Mn elements in EDX analyses (Fig. S11).

Apart from the Mn dissolution, the key point is to illuminate the mechanism of MnO_2_-based Zn ions battery. Ex-XRD characterization was carried out to trace the phase changes of the cathode during the charge–discharge process. A sharp peak at around 10° (2 thetas) is indexed to ZnSO_4_·3Zn(OH)_2_·*n*H_2_O (*n* = 3 with JCPDS No. 78–0247) (Fig. 3d), which illustrates the formation of massive basic zinc sulfate at the surface of MnO_2_ cathode in the liquid electrolyte at discharge states of 1.2, 1.0, and 0.8 V, respectively. On the contrary, no obvious new peaks are observed at around 10° for the same state of discharge in the HCCE system, which means less by-products formation at the discharge/charge states [33, 37]. which is confirmed by the clean surface morphology of SEM images based on HCCE under corresponding voltage (discharge state of 1.0 and 0.8 V, Figs. 3b, c and S12). To be summarized, the superior performance of Zn/MnO_2_ battery based on HCCE as the formation of a protective layer surrounding MnO_2_ materials, which is mainly caused by the suppression of Mn dissolution and irreversible basic zinc sulfate.

Also, the Zn^2+^ ion diffusion coefficients was in-depth analyzed by galvanostatic intermittent titration technique (GITT) technology and CV experiment. It confirmed that the cell with HCCE and the liquid ones exhibited similar electrochemical reaction kinetics, e.g., Zn^2+^ ion diffusion coefficients (Fig. S13), capacitive contribution (Fig. S15) and ionic conductivity (HCCE: 1.1 × 10^–2^ S cm^−2^, liquid: 0.92 × 10^–2^ S cm^−2^) (Fig. S16). This means that Zn^2+^ can pass through the film easily without obstruction. Next, the influence of HCCE on the anode will be discussed.

### Analysis of Anode of Zn/MnO_2_ Battery

The plating/stripping stability of Zn cycled with HCCE was undertaken by the galvanostatic measurements in Zn/Zn symmetrical battery at the three different current densities (0.28, 1.13, and 2.83 mA cm^−2^, respectively) as shown in Fig. S17a. The HCCE battery has smaller voltage fluctuations without obvious polarization in comparison with the liquid one. Long-time plating/stripping stability of the Zn/Zn battery is confirmed by the stable voltage–time curves with 29.8 mV voltage hysteresis at the end of 400 h (Fig. S17b). The stability may deduce the facility of charge transference as shown in Fig. S17c. Besides, the plating/stripping reversibility of Zn in the HCCE was investigated by using cyclic voltammetry (CV) measurement for Zn–Zn symmetrical battery. As shown in Fig. S18, the cell with HCCE shows better reversibility of 99% (cathodic) than that of liquid one (65%) at a scan rate of 2 mV s^−1^, which is similar to that of the high-concentration 1 m Zn(TFSI)_2_ + 20 m LiTFSI-based electrolytes [17].

In order to get further insight into the interfacial charge transfer process, the activation energies (*E*_*a*_) representing the journey of Zn^2+^ were obtained through fitting the semicircles (*R*_*s*_, *R*_ct_) in Zn-Zn symmetrical batteries (Fig. S19a, b) at different temperatures from 0 to 40 ℃ and the *E*_*a*_ were related to the environment, temperature, etc. This is becasue Abe and co-workers have confirmed that the transport of Li through the SEI and charge transfer showed a close dependence on temperature [38, 42]. *E*_a1_ represents the transport process of Li^+^. *E*_a2_ exhibits the de-solvation energy of Li^+^ that has been widely accepted as the dominant barrier in the electrochemical process [39, 40]. The dissolution of zinc ions at the anode interface is restricted by the structure of the solvated sheath and the double electric layer, the interfacial charge transfer behavior is highly similar to that of lithium ion [41, 42]. The *E*_*a1*_ and *E*_*a2*_ values are tested under the same conditions, both of *E*_*a1*_ and *E*_*a2*_ are in line with the law of Arrhenius:$\frac{1}{{R}_{\mathrm{ct}}}=A \mathrm{exp}(-{E}_{a}/\mathrm{RT})$. As a result of the calculation (Fig. S19c, d), *E*_*a1*_ is 4.5 kJ mol^−1^ and *E*_*a2*_ is 32.3 kJ mol^−1^ in the HCCE, smaller than that of liquid electrolyte (*E*_*a1*_ is 10.7 kJ mol^−1^ and *E*_*a2*_ is 52.5 kJ mol^−1^). Compared with liquid electrolytes, the smaller charge transfer activation energy exhibits the easier transfer of solvated zinc ion between the Zn anode and the HCCE interface, which is consistent with EIS results for Zn-Zn symmetric batteries (Fig. S17c). Besides, as for the reveal of high ions conductivity, the distribution of pore diameter for the HCCE (dry) is mainly concentrated on 3.6 nm range and no other large holes (Fig. S20), which may lead to the conducive channel to confine Zn^2+^ diffusion and the massive charge transference. So as to avoid dendrite growth caused by excessive Zn^2+^ accumulate on local at the zinc anode surface with available charge transference capacity. Therefore, it can be confirmed by the SEM results that the Zn anode maintained a relatively flat and smooth surface without obvious dendrite (or accumulation) in the presence of the HCCE after 200 cycles at 1000 mA g^−1^ (Fig. 3e), while rough aggregation and lots of remainings with hexagon layer structure were observed for liquid ones (Figs. 3f and S21a), which are further confirmed by the more uniform surface atomic force microscope (AFM) in HCCE system as shown in Fig. 3g. As for the hexagon layer structure, many researchers blame it on the partially irreversible Zn-based layered double hydroxides (Zn LDHs), which is easy to detach from the electrode surface [43, 44]. Large amounts of Zn LDHs are randomly distributed and accumulated on the surface of zinc anode cycled with liquid (Figs. 3f and S21a), which makes the surface uneven (Fig. 3h). However, no Zn LDHs products appeared on the Zn anode cycled with HCCE (Figs. 3e and S21b, c), and the surface remained smooth (Fig. 3g). Along with concomitant precipitation of a Zn LDH, the electrochemically insert by-products will passivate the electrode surface. In our system, Fig. S22 reveals the XRD pattern of the corresponding Zn anode after 200 cycles. For the HCCE system, no obvious new peaks are observed at around 11° well-indexed to the zinc sulfate hydroxide hydrate of 6Zn(OH)_2_·ZnSO_4_·4H_2_O (JCPDS No. 11-0280), suggesting the side-reaction can be largely suppressed by HCCE. Besides the limited amount of free water, the battery cycled with HCCE showed two distinct half-circles (Fig. S17c), indicating the presence of interfacial impedance, confirming that the HCCE coated the electrode to form a protective layer. Therefore, the introduction of HCCE not only avoids the direct contact between metal Zn and free H_2_O by the formation of protective layer with good ions conductivity to reduce the irreversible products along with Zn^2+^ plating/stripping process on the anode surface, but also decreases the de-solvation barrier, facilitating the charge transfer of Zn^2+^.

On the other hand, corrosion of Zn anode and deposition model has also carried out for HCCE group compared to liquid one, respectively. The Zn corrosion was analyzed by a linear polarization test. Compared with the liquid electrolyte, the HCCE had a lower corrosion current (Fig. S23a), illustrating the lower corrosion rate [45]. The variation in current–time curves at a constant potential can sensitively reflect the nucleation process and surface change [46, 47]. For the HCCE group, Fig. S23c shows that it only takes 40 s to reach current equilibrium for Chronoamperometry (CA) result, and remains almost constant with lower current density ( 13.66 mA cm^−2^) than that of liquid one ( 24.75 mA cm^−2^) at 200 mV for 300 s that applied, which leads to much smooth surface morphology cycled with HCCE [45, 48]. Generally, to minimize the surface energy and the exposed area, Zn^2+^ ions tend to aggregate and grow into dendrites. The nucleation overpotential of Zn/Cu cells cycled with HCCE is only 43 mV (Fig. S23d), which increases the nucleation active sites of Zn^2+^ ions and the nucleation positions.

### Mechanism Analysis of Aqueous Zn/MnO_2_ Battery with Colloidal Electrolyte

**Fig. 4:**
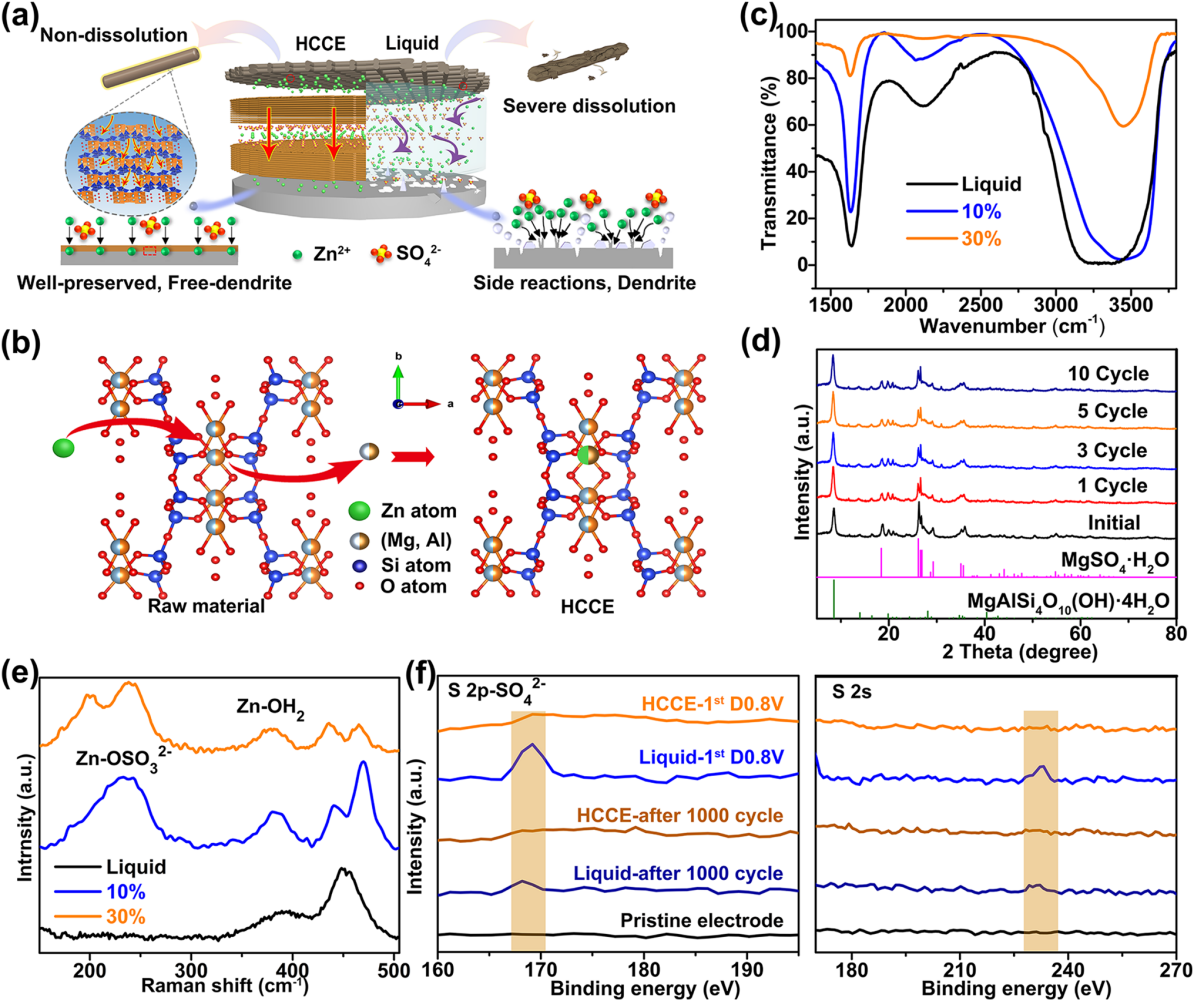
**a** Schematic diagram of interface protection effect in HCCE and liquid electrolyte. **b** Ion exchange diagrams in the HCCE. **c** FTIR spectra (1400–3800 cm^−1^) of colloidal electrolyte with different concentration and liquid electrolyte. **d** XRD patterns of HCCE (after drying) with different cycles: initial (no cycle), 1 cycle,3 cycles, 5 cycles, 10 cycles. **e** Raman spectra of colloidal electrolyte and liquid electrolyte. **f** XPS spectrum image of the cathode of the battery with HCCE and liquid electrolyte after initial discharge and 1000 cycles

Discussed above, the schematic diagram of interface protection effect in HCCE and liquid electrolyte as shown in Fig. 4a. The HCCE can form the protective layer to protect both cathode and anode of aqueous Zn-ion battery. The protective layer can effectively inhibit the formation of irreversible electrode surface hydroxyl zinc sulfate, and it will not hinder the zinc ions transport compared with liquid. More importantly, it not only can inhibit the dissolution of manganese and corrosion of anode, lower de-solvation energy, and more nucleating active site can also maintain the Zn^2+^ continues for a long-time high reversible deposition/stripping reaction, to obtain the well-preserved electrode.

For the HCCE, Fig. 4c shows the FTIR spectra of colloidal electrolyte with various concentrations. As the concentration of palygorskite increases, the peak at about 1630 cm^−1^ (δ$_{{{\text{H}}_2}{\text{O}}}$) decreases, while the peak at about 3419 cm^−1^ (υ$_{{{\text{H}}_2}{\text{O}}}$) decreases with hypsochromic shift (HCCE: 3449 cm^−1^ (υ$_{{{\text{H}}_2}{\text{O}}}$), liquid: 3419 cm^−1^ (υ$_{{{\text{H}}_2}{\text{O}}}$)). Attributing it to the polar-silica-hydroxyl of palygorskite forms hydrogen bonds with water molecules in the HCCE, which exerts a polar effect on water molecules and strengthens hydrogen bonds, resulting in peak (υ$_{{{\text{H}}_2}{\text{O}}}$) blueshift. A large amount of free water is converted into adsorbed water of palygorskite [49], which slow the decomposition of free water in the electrolyte (similar to the high-concentration 1 M Zn(TFSI)_2_ + 20 M LiTFSI-based electrolytes [17]) making the decomposition voltage of HCCE (1.60 V) higher than that of liquid (1.56 V) (Fig. S23b). In Fig. 4e, as the concentration increases, the Raman band (~ 240 cm^−1^) is attributed to [Zn^2+^·OSO_3_^2−^] ligand mode [50]. Amplification peaks of [Zn^2+^·OSO_3_^2−^] ligand mode means a more constrict complex ion, which seem impossible in ZnSO_4_ solutions because limited solubility. Raman spectroscopy confirmed that palygorskite can remove a large number of water molecules to achieve the effect of super-saturated electrolyte and limit the solvation of Zn^2+^.

In order to further explore the way of Zn^2+^ passing through the HCCE, we speculate that palygorskite will spontaneously inhale and release Zn^2+^ according to the concentration gradient in the electrolyte. The HCCE (wash) is obtained by mixing HCCE with distilled water (dilute the Zn^2+^ concentration), centrifugation, and drying. The corresponding XRD (Fig. S24a) displays that the phase of MgSO_4_·H_2_O disappears, and then returns to the pristine phase of the raw palygorskite. And after 3, 5, and 10 cycles, the XRD peaks of HCCE (Fig. 4d) almost same with initial (no cycle) which shows that the HCCE has good stability. As shown in the infrared spectrum at ~ 3585 cm^−1^ (υ$_{{\text{Mg}}_{2}-{\text{OH}}}$) and ~ 3615 cm^−1^ (υ_(Mg, Al)-OH_) (Figs. 1b and S24b), the (υ$_{{\text{Mg}}_{2}-{\text{OH}}}$) and (υ_(Mg, Al)-OH_) peak appears again. These results demonstrate that with the change of the concentration of zinc ions in the solvent, in order to bring the concentration of zinc ions in palygorskite and zinc ions in the solvent into a new dynamic equilibrium, the zinc ions and magnesium ions in palygorskite will spontaneously exchange with the zinc ions and magnesium ions in the solvent (Fig. 4b). Eventually, palygorskite will be spontaneously inhaled and release Zn^2+^ according to the concentration gradient in the electrolyte.

The XPS tests were carried out on the cathode cycled with HCCE and the liquid electrolyte after the first discharge state of 0.8 V and 1000 cycles, respectively. As shown in Fig. 4f (the full spectrum is shown in Fig. S25), two distinct peaks at 168.5 and 230 eV, representing the aggregation of SO_4_^2−^, are obviously observed in the electrode cycled with liquid electrolytes for both initial discharge and 1000 cycles, whereas the HCCE is absent of these signals. It suggests that the HCCE is capable of inhibiting the accumulation of anions on the surface, which may be due to the relatively higher intrinsic negative charges of palygorskite, the SO_4_^2−^ anions were repulsed away from surfaces [49, 51], further explaining why the electrode surface can inhibit the formation of zin hydroxy sulfate. Meanwhile, the higher Zn^2+^ transference number of the HCCE (t_Zn_^2+^  ~ 0.64 compared to ~ 0.50 for liquid ones) (Fig. S26) based on the same ionic conductivity between HCCE and liquid. It indicates that more Zn^2+^ ions are involved in effective migration in the HCCE, which is attributed to the limited migration of anions (SO_4_^2−^) [52]. In this way, the presence of Zn^2+^ ions within the palygorskite is protected, and the solvation effect can be reduced. Based on the lower de-solvation energy of the HCCE, Zn^2+^ ions can pass through this “protective layer” quickly and making it easier for Zn^2+^ ions to deposit near the initial adsorption of the Zn anode, thus significantly inhibiting the formation of irreversible product (such as basic zinc sulfate) and dendrite growth.

## Conclusion

In summary, the HCCE can form the protective layer to protect both the cathode and anode of an aqueous Zn-ion battery. For the cathode, the presence of the protective film can inhibit the dissolution of manganese element and the formation of irreversible products. For the anode and electrolyte, uniform pore size distribution and lower de-solvation energy accelerate the migration and charge transfer of Zn^2+^. The HCCE has higher ionic conductivity, can stabilize the zinc stripping/deposition process and inhibit the corrosion as well as the growth of zinc dendrites, thus obtaining good electrochemical performance. This discovery may bring profound impacts toward the development of high-performance aqueous rechargeable battery by providing a facile and effective strategy for life cycle improvement.

## Supplementary Information

Supplementary file1 (PDF 15 kb)
