# Supplementary material for: Inorganic Colloidal Electrolyte for Highly Robust Zinc-Ion Batteries
Source: Nanomicro Lett. 2021 Feb 11;13:69. doi: 10.1007/s40820-021-00595-6 (PMC8187543; doi:10.1007/s40820-021-00595-6)
Supplement: Supplementary file 1 — Supplementary file1 (PDF 15 kb) [file 40820_2021_595_MOESM1_ESM.pdf]

Supporting Information for

Inorganic Colloidal Electrolyte for Highly Robust Zinc-Ion Batteries

Jiawei Gao[1, #], Xuesong Xie[1, #], Shuquan Liang[1, 3], Bingan Lu[2, *], Jiang Zhou[1, 3, *]

[1]School of Materials Science and Engineering, Central South University, Changsha 410083, P. R. China

[2]School of Physics and Electronics, Hunan University, Changsha 410082, P. R. China

[3]Key Laboratory of Electronic Packaging and Advanced Functional Materials of Hunan Province, Central South University, Changsha 410083, P. R. China.

[#]Jiawei Gao and Xuesong Xie contributed equally to this work.

*Corresponding authors. E-mail: zhou_jiang@csu.edu.cn (Jiang Zhou); luba2012@hnu.edu.cn (Bingan Lu)

Supplementary Figures and Tables

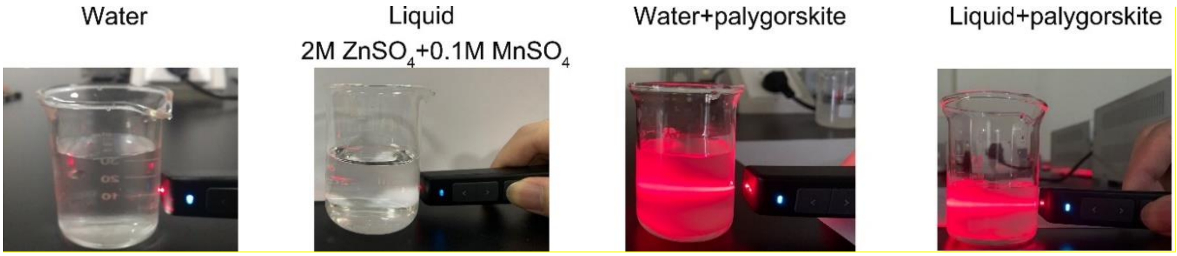

Fig. S1 Tyndall effect in water, liquid, water + palygorskite and liquid + palygorskite

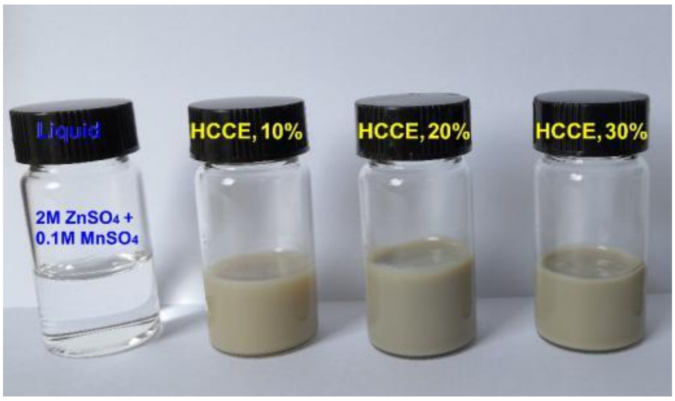

Fig. S2 Photographic images of liquid electrolyte and concentration colloidal electrolyte (HCCE, 10 %, 20 % and 30 %)

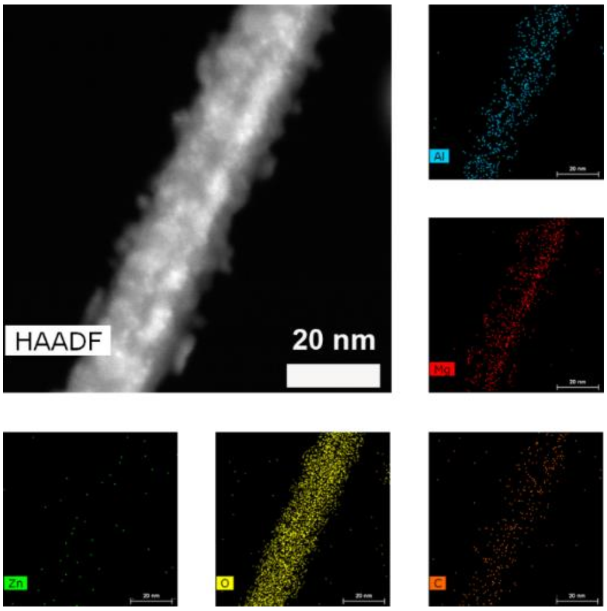

Fig. S3 TEM micrograph with EDX elemental (Mg, Al, Zn, O, C) mapping images of raw material powders (Palygorskite)

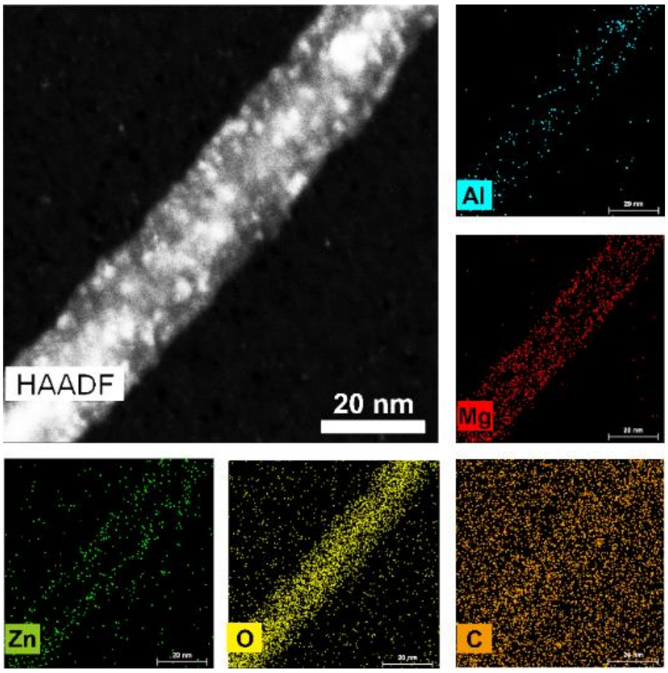

Fig. S4 TEM and EDX elemental (Mg, Al, Si, O, Zn) mapping images of colloidal electrolyte (after drying)

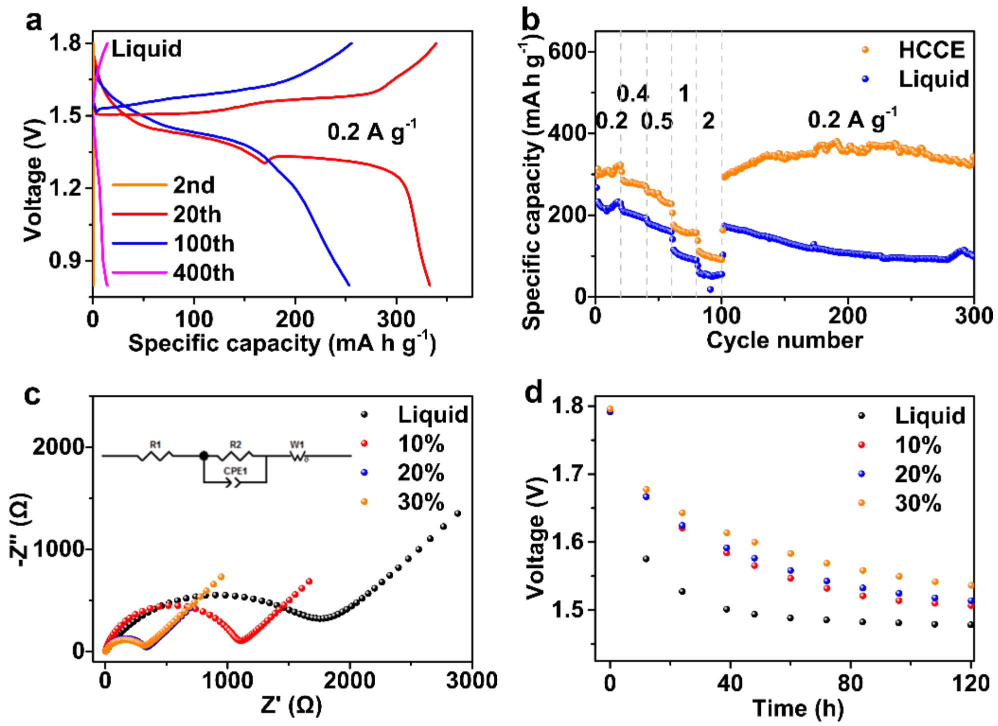

Fig. S5 (**a**) Galvanostatic charge-discharge curves of the different cycles at 0.2 A g^{-1} of the cell with liquid electrolyte. (**b**) Rate capability of the cell with HCCE and liquid electrolyte from 200 to 2000 mA g^{-1}. (**c**) Nyquist plots, equivalent circuit and fitted results of different colloidal concentrations electrolyte and liquid electrolyte based on Zn-MnO_2 cells (an ohmic internal resistance and a charge-transfer resistance shown in **Table S1**). (**d**) Open circuit potential decays for the different concentration colloidal electrolyte and liquid electrolyte after the conditions (charging and discharging at current density of 200 mA g^{-1} during two cycle, then charging at current density of 100 mA g^{-1} to 1.8V, and holding at this voltage for 24 h)

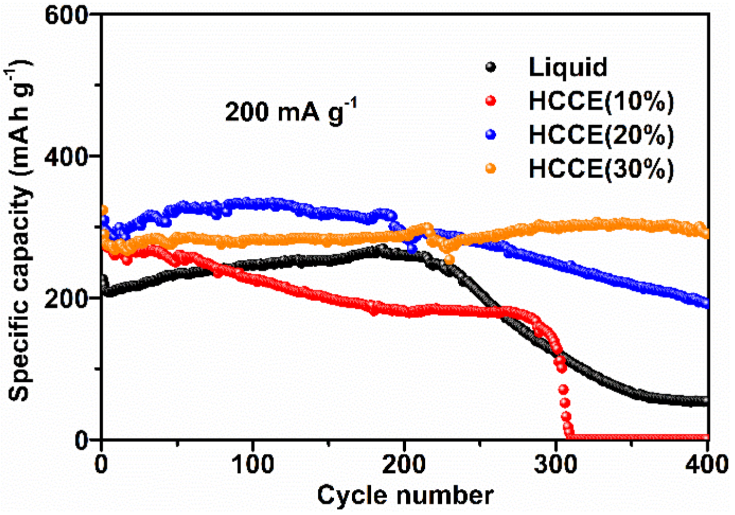

Fig. S6 Different of electrochemical performance between the colloidal electrolyte with different concentration

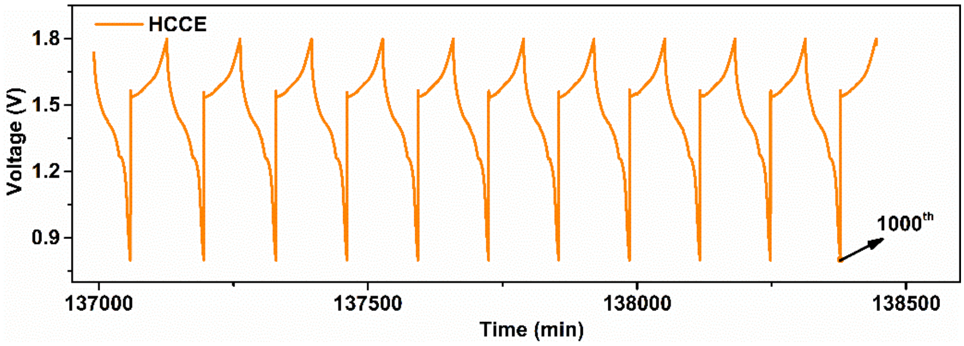

Fig. S7 Last ten charge/discharge curves of the cell with HCCE at 0.5 A g^{-1}

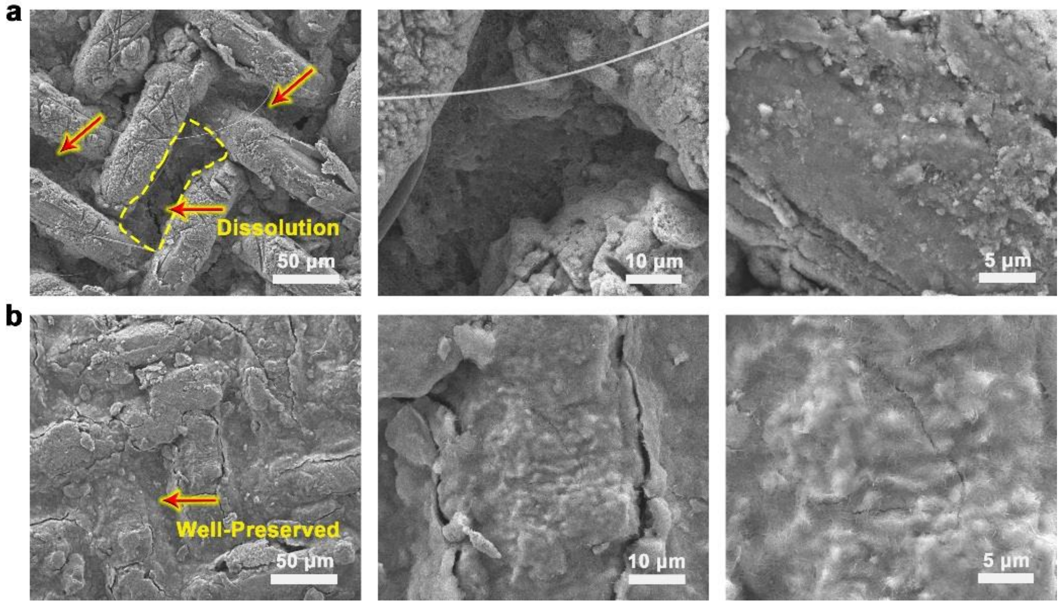

Fig. S8 SEM images of the cathode of the battery after 200 cycles at 1 A g^{-1} with (**a**) Liquid electrolyte and (**b**) the HCCE

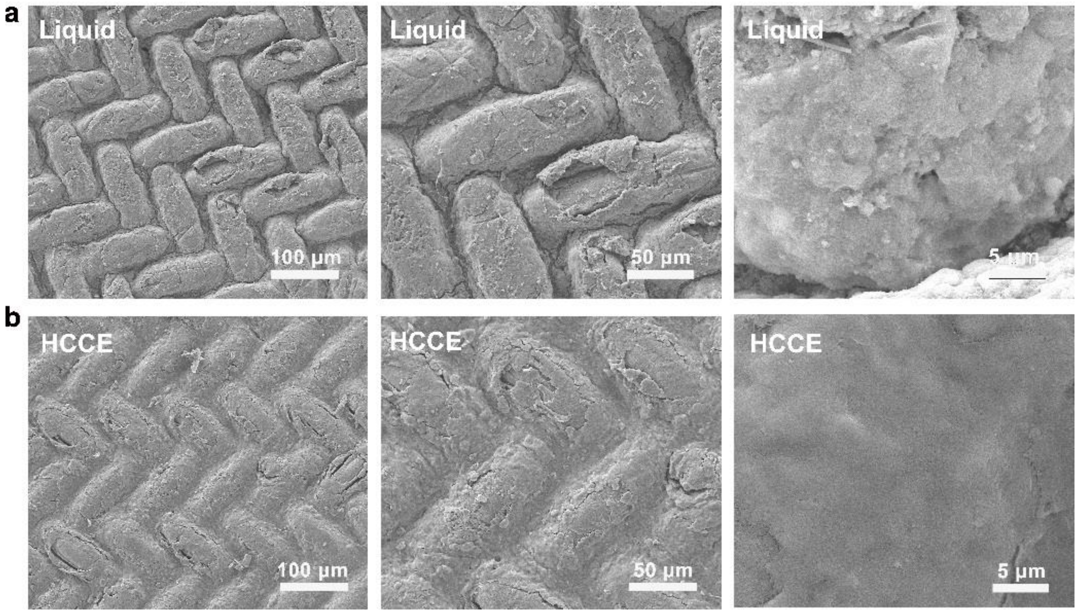

Fig. S9 SEM images of the cathode of the battery after 400 cycles at 0.2 A g^{-1} with (**a**) Liquid electrolyte and (b) the HCCE

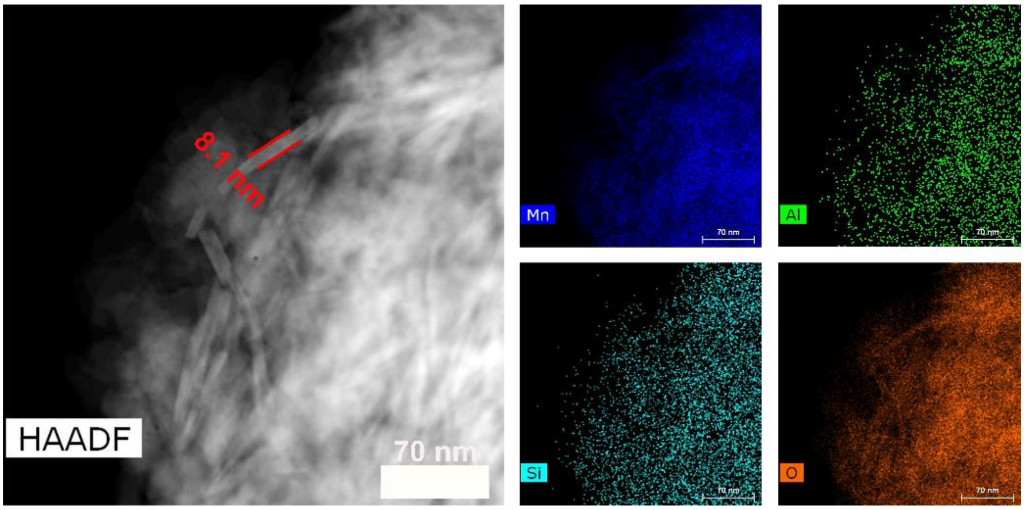

Fig. S10 TEM and EDX elemental (Mg, Mn, O, Zn) mapping images of cathode with liquid after initial fully discharge to 0.8 V at 200 mA g^{-1}

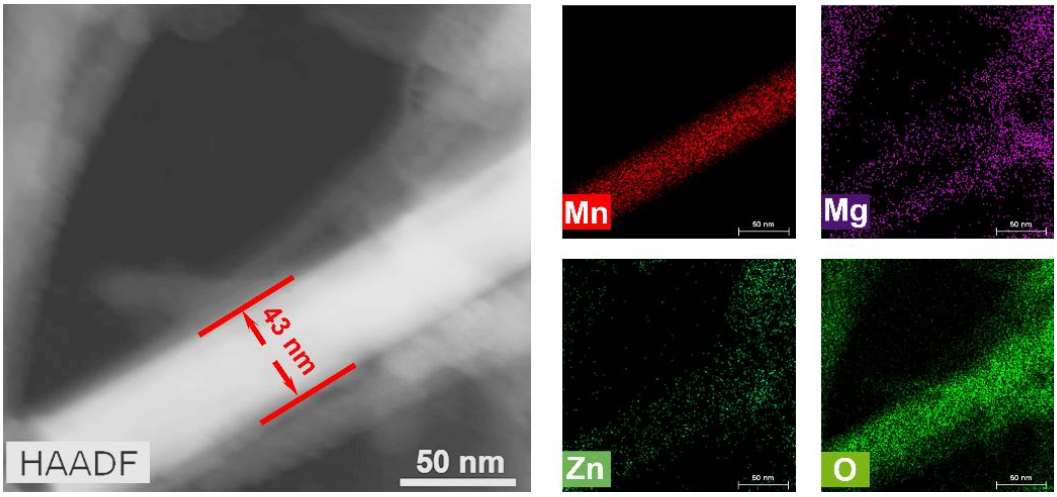

Fig. S11 TEM and EDX elemental (Mg, Mn, O, Zn) mapping images of cathode with HCCE after initial fully discharge to 0.8 V at 200 mA g^{-1}

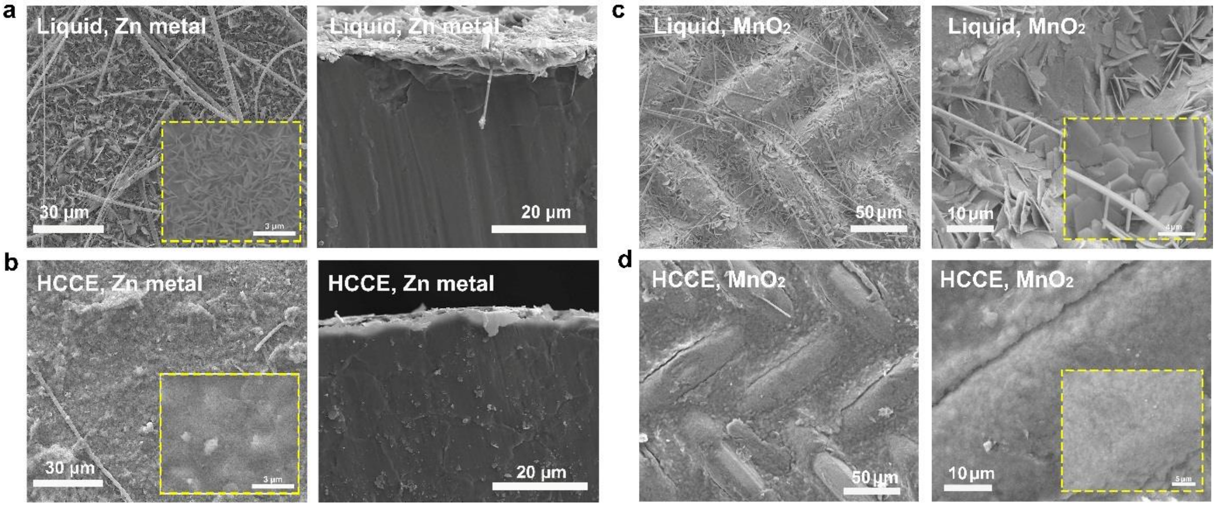

Fig. S12 SEM images of 1st fully discharge to 0.8V: on the anode of the cell with (**a**) Liquid electrolyte and (**b**) the HCCE. on the cathode of the cell with (**c**) Liquid electrolyte and (**d**) the HCCE

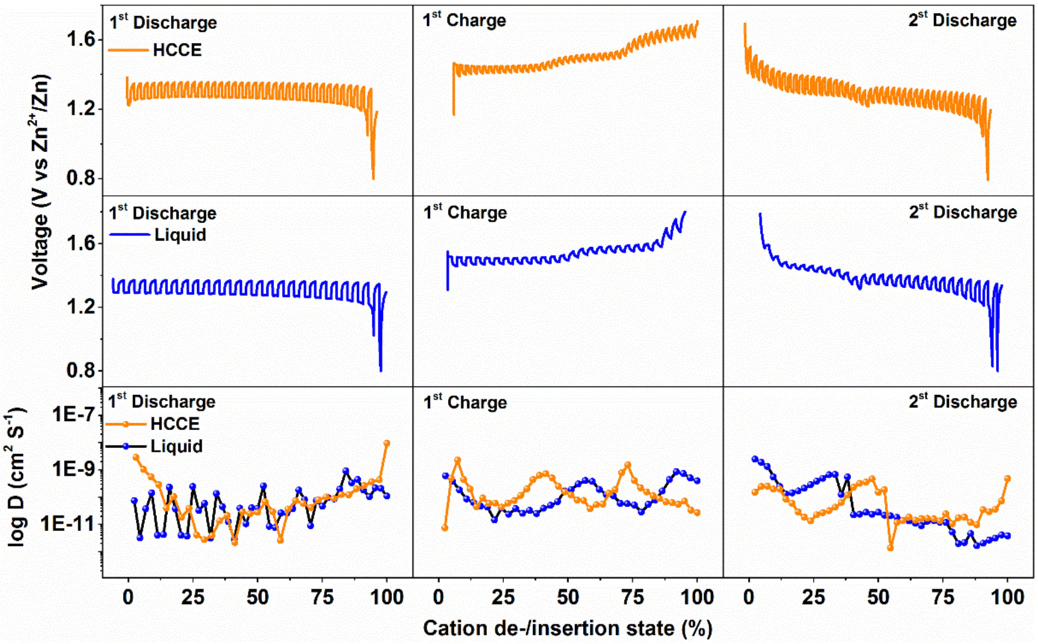

Fig. S13 GITT and diffusion coefficient contrast curve of the Zn/α-MnO_2 cells with HCCE and Liquid electrolyte (The detail calculation process is listed in the **Fig. S14**)

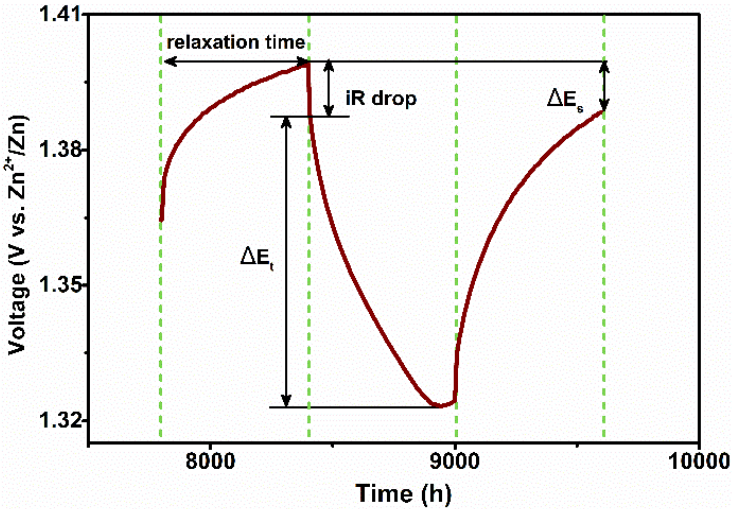

Fig. S14 *E vs. t* curves of the MnO_2 electrode cycled with HCCE and liquid electrolyte for a single GITT during discharge process. The diffusion coefficient was conducted by using Galvanostatic Intermittent Titration Technique (GITT) and calculated based on Eq. as follows [S1]:

$$D = \frac{4L^2}{\pi t}\left(\frac{\Delta E_S}{\Delta E_t}\right)^2$$

Where t is the duration of the current pulse (s), ΔEs is the steady-state potential change (V) by the current pulse. ΔEt is the potential change (V) of the constant current pulse excluded the iR drop. L is ion diffusion length (cm); for compact electrode, it is equal to the thickness of the electrode.

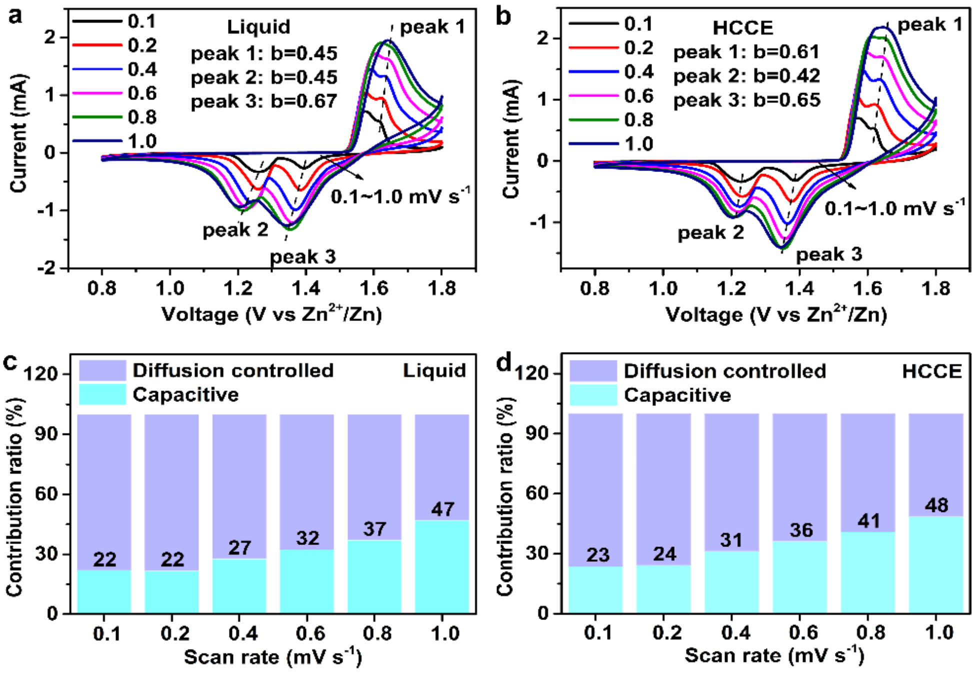

Fig. S15 (**a**) CV curve of the cell with Liquid electrolyte at different scan rates; (**b**) CV curve of the cell with HCCE at different scan rates; (**c**) capacitance contribution ratio of the cell with Liquid electrolyte at different scan rates; (**d**) capacitance contribution ratio of the cell with HCCE at different scan rates

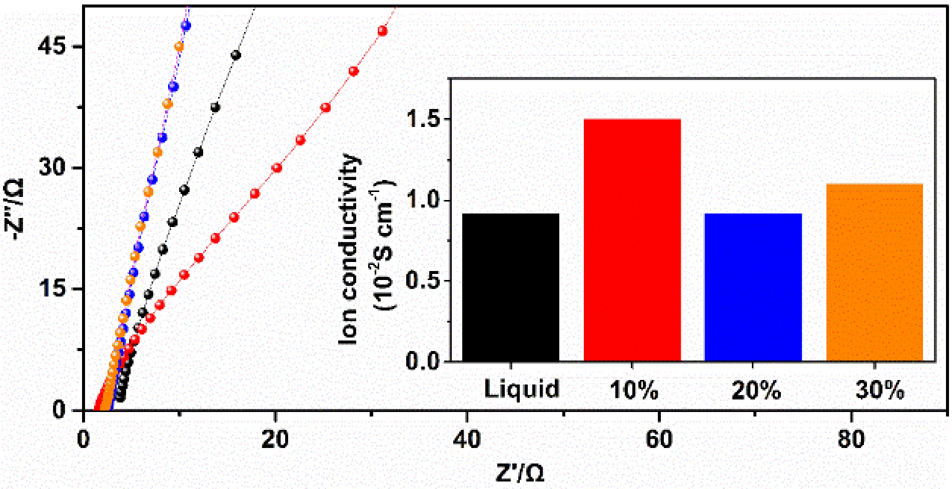

Fig. S16 AC impedance spectra of the different concentration HCCE and liquid electrolyte in the frequency range from 10 kHz to 0.01 Hz, inset: ion conductivity of different concentration HCCE and liquid electrolyte. The corresponding ionic conductivity was calculated by [S2]: $\sigma = \frac{l}{RA}$ where l is the thickness of gel electrolyte, R is the bulk resistance, A is the contact area of electrolyte.

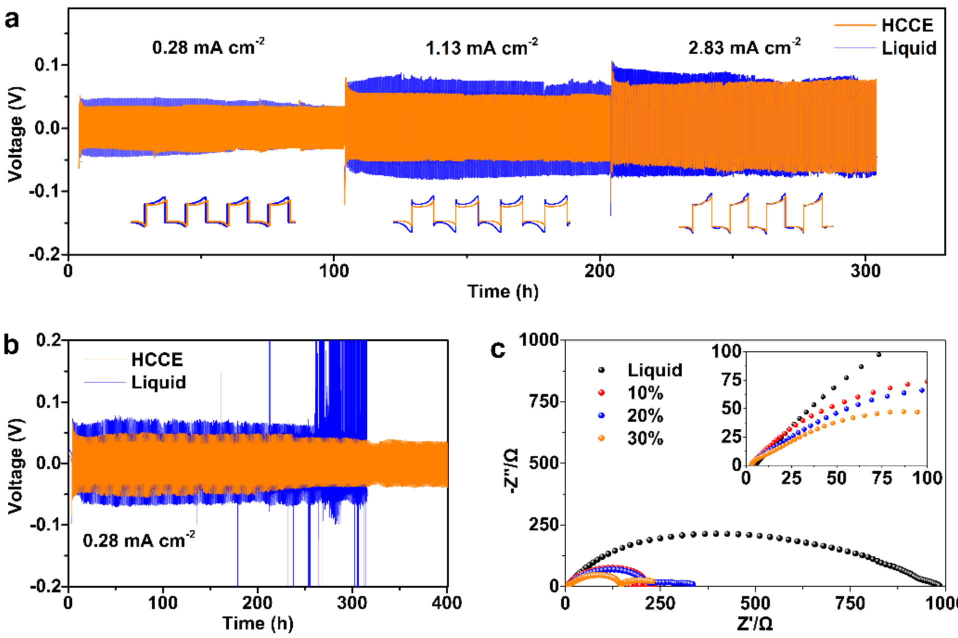

Fig. S17 Based on Zn-Zn symmetrical cells: (**a**) Cyclic plating/stripping process with the HCCE and liquid electrolyte at different constant current density of 0.28, 1.13, and 2.83 mA cm^{-2}. (**b**) Cyclic plating/stripping process with the HCCE and liquid electrolyte at current density of 0.28 mA cm^{-2}. (**c**) Nyquist plots, equivalent circuit and fitted results of different concentration Colloidal electrolyte and Liquid electrolyte (an ohmic internal resistance and a charge-transfer resistance shown in **Table S2**)

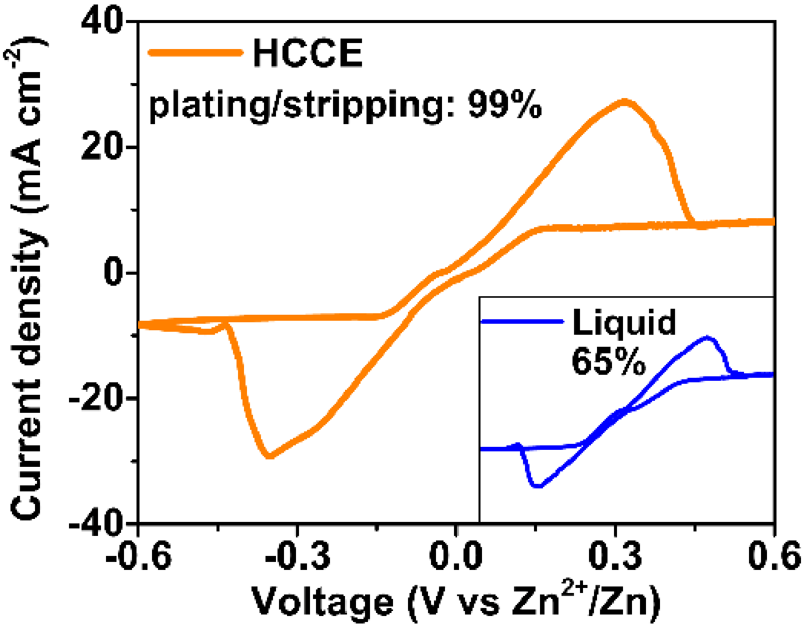

Fig. S18 Cyclic voltammogram of Zn plating/stripping in Zn-Zn symmetric cells with HCCE at a scan rate of 2 mV s^{-1} (in the third cycle). Inset: Cyclic voltammetry in liquid electrolyte under the same conditions

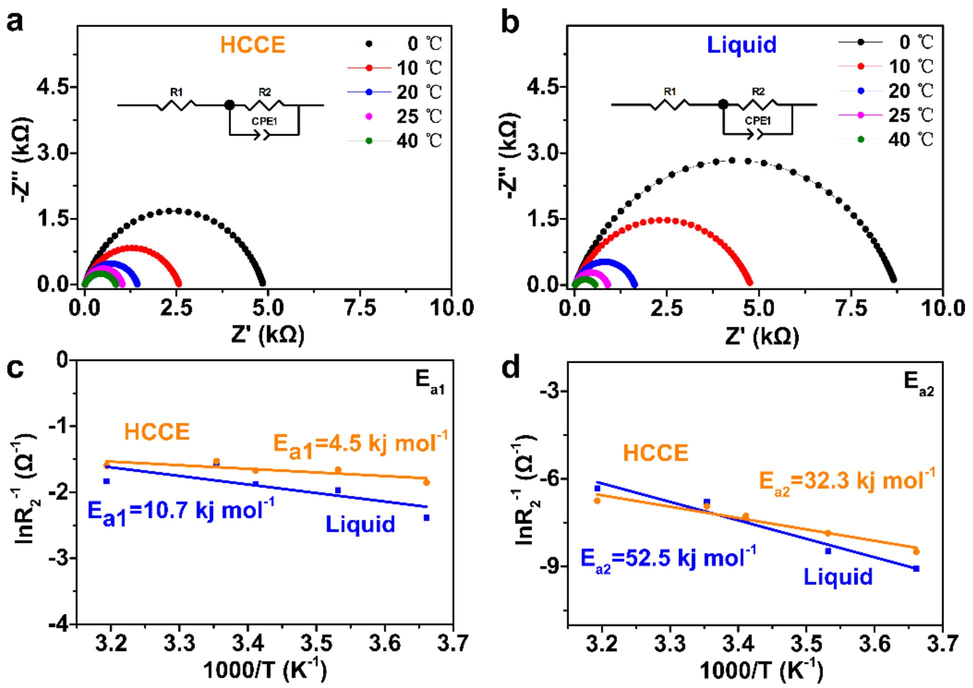

Fig. S19 (**a**) and (**b**) Nyquist plots of the Zn/Zn symmetric cells at different temperatures (an ohmic internal resistance and a charge-transfer resistance shown in **Table S5**). (**c**) and (**d**) Arrhenius behavior and comparison of activation energies between the *Rs* and *Rct* derived in Nyquist plots of Zn/Zn symmetrical cells

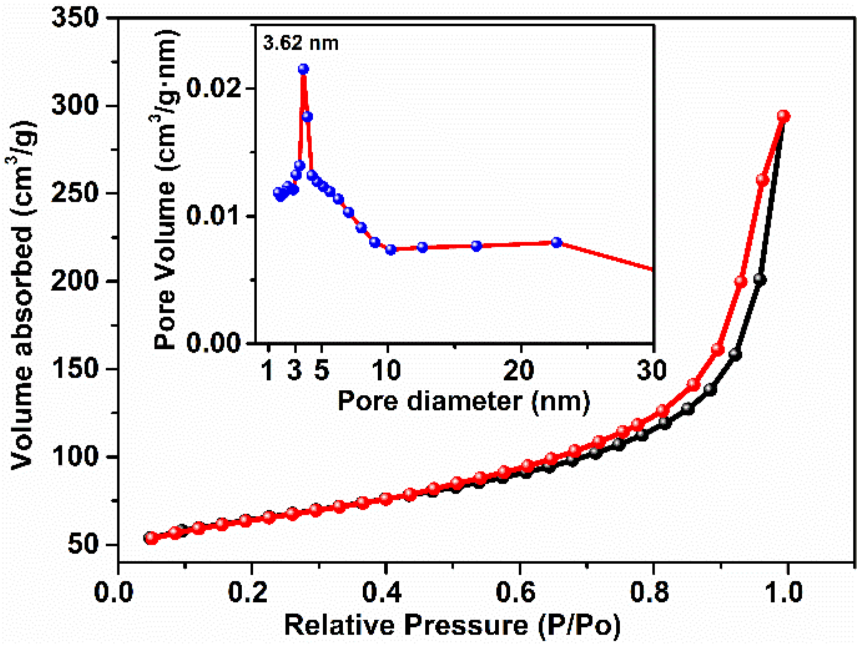

Fig. S20 Absorption and desorption curves (inset: the aperture distribution) of the raw material (Palygorskite)

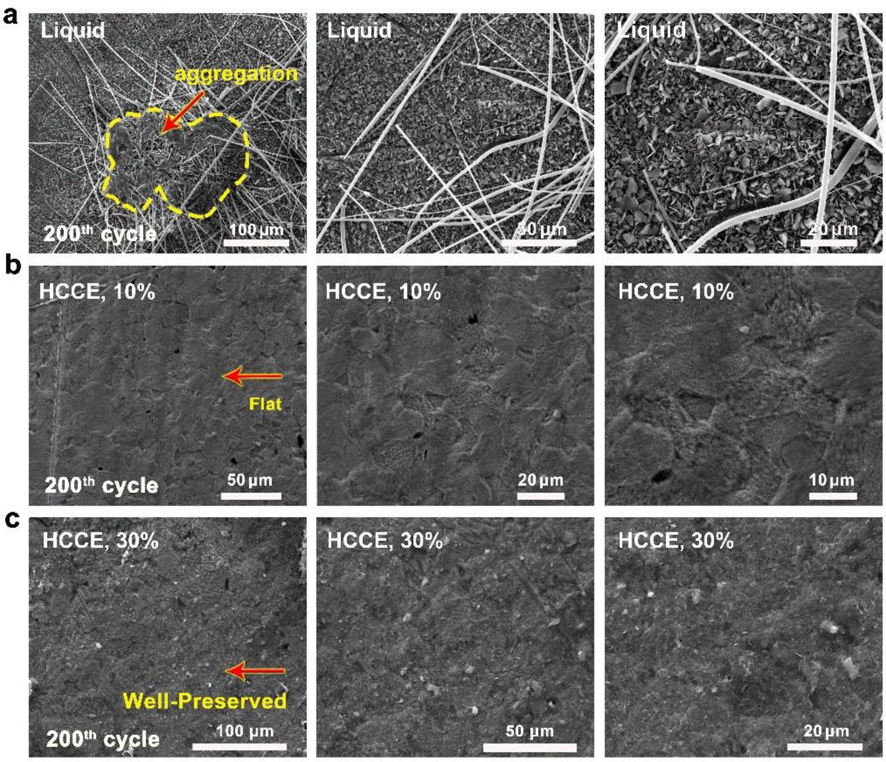

Fig. S21 SEM images of anode of the battery after 200 cycles at 1000 mA g^{-1} with (**a**) liquid electrolyte, (**b**) HCCE (10%), (**c**) HCCE (30%)

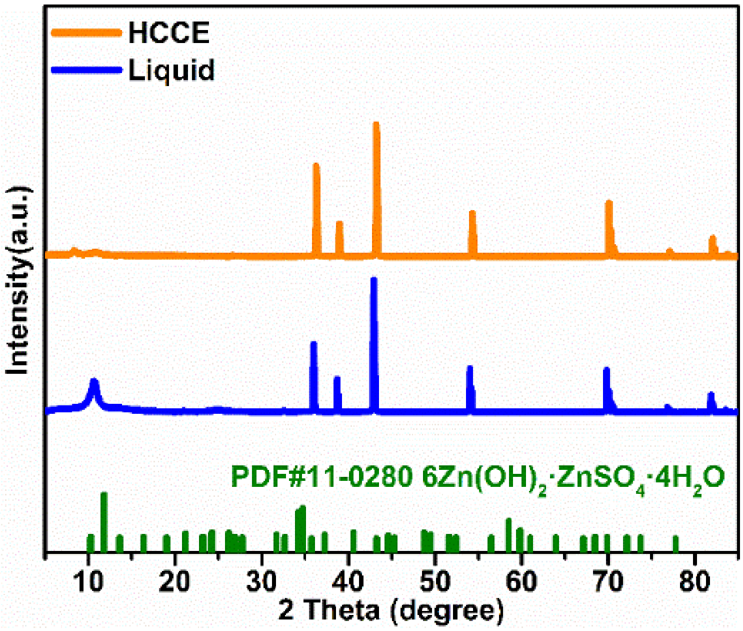

Fig. S22 The ex-situ XRD patterns of cathode cycled over 200 cycles at 1000 mA g^{-1} of the cell with the HCCE and liquid ones

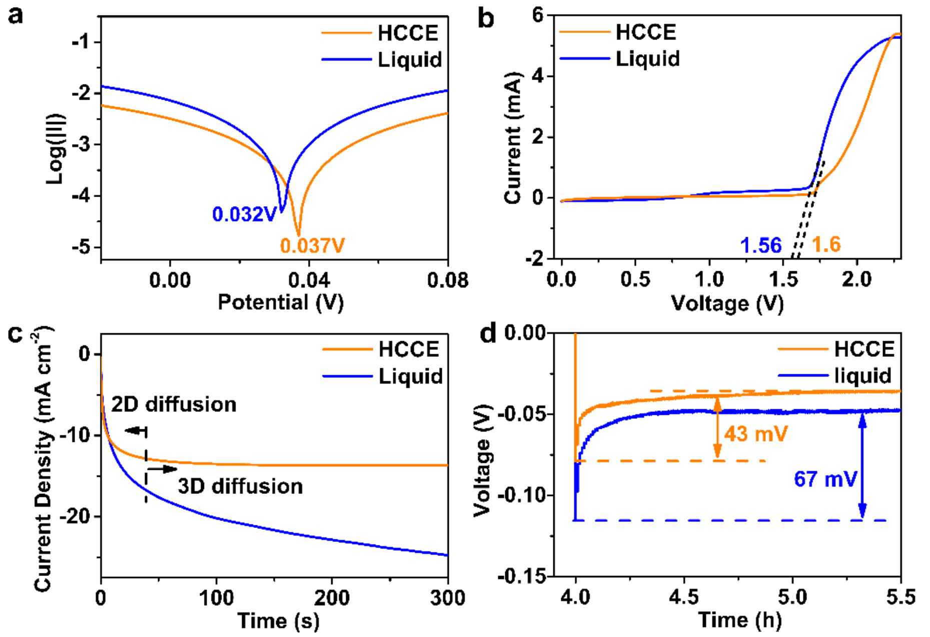

Fig. S23 (**a**) Linear polarization curves (Scan rate: 0.01 V s^{-1}) and (**c**) Chronoamperometry of zinc when in contact with different electrolytes based on Zn-Zn symmetrical cells at a overpotential of -200 mV for 300 s that applied, (**b**) Linear Sweep Voltammetry (LSV) curves based on Zn-SSWM cells, (**d**) voltage-time curves during Zn nucleation at 2 mA cm^{-2} of the Zn/Cu cells with HCCE and liquid electrolyte

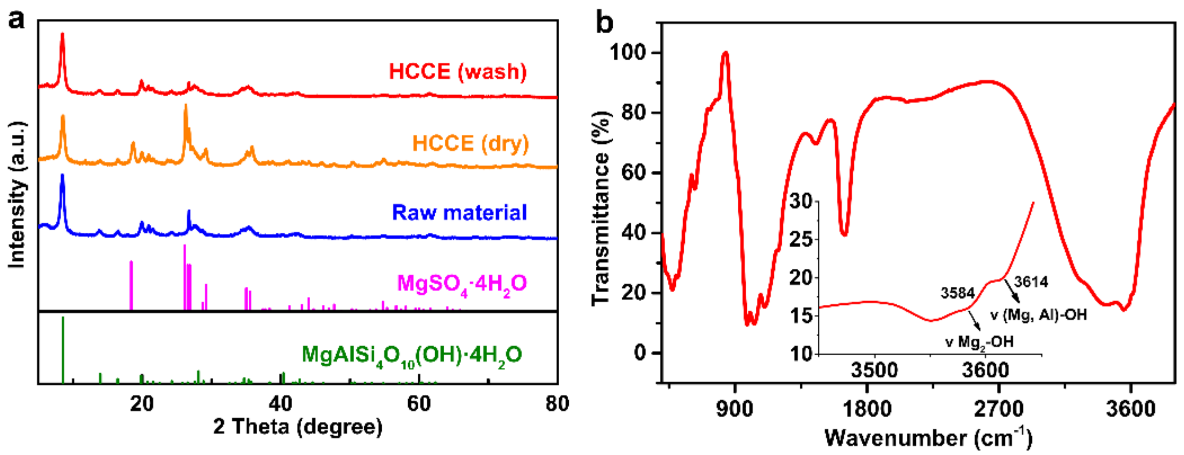

Fig. S24 (**a**) ex-situ XRD and (**b**) FTIR spectra of the HCCE washed by deionized water

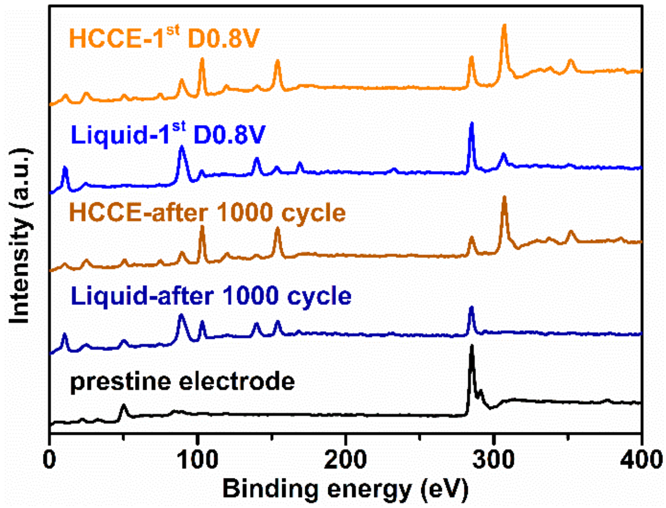

Fig. S25 Full XPS spectrum image of the cathode of the battery with HCCE and liquid electrolyte after initial discharge and 1000 cycles

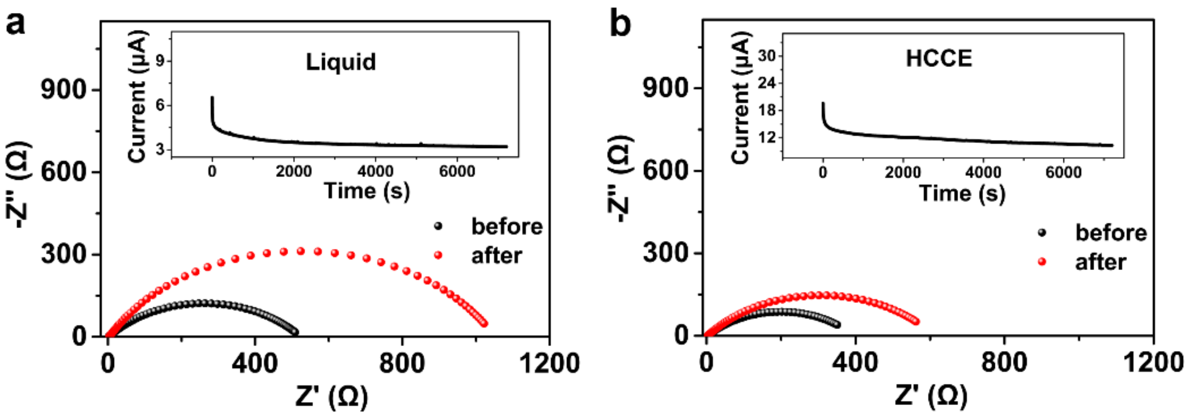

Fig. S26 EIS of the Zn-Zn symmetric cell (a) with liquid electrolyte and (b) HCCE before and after polarization (an ohmic internal resistance and a charge-transfer resistance shown in **Table S3**), inset: variation of current with time during polarization at an applied voltage of 5 mV at room temperature. The values of t_+ were calculated using the following equation [S3]: $t_+ = \frac{I_s(\Delta V - I_0 R_0)}{I_0(\Delta V - I_S R_S)}$

Where I_0 and I_s refer the initial and steady-state current, ΔV is the potential applied across the cell, R_0 and R_s are, respectively, the initial and steady-state resistances of the passivation layer on the Zn electrode determined by impedance spectroscopy (the specific data shown in **Table S4**).

Table S1 Impedance fitting parameters of different concentration of Colloidal electrolyte Zn/MnO_2 cells before cycling

	Liquid		10%		20%		30%	
	**Value**	**Error (%)**	**Value**	**Error (%)**	**Value**	**Error (%)**	**Value**	**Error (%)**
Rs	3.2	7.91	1.3	4.69	3.1	0.06	2.1	3.15
Rct	1697	2.77	1058	1.62	313	4.04	299	1.61
W_1	$1.9E^{-5}$	7.93	$1.3E^{-6}$	6.58	$8.4E^{-7}$	3.02	$1.4E^{-6}$	2.61

Table S2 Impedance fitting parameters of different concentration of Colloidal electrolyte Zn/Zn symmetric cells before cycling

	**liquid**	**10%**	**20%**	**30%**
Rs	6.057	3.286	2.892	2.855
Rct	984.3	217.9	224.5	145.3

Table S3 Impedance fitting parameters of different concentration of Colloidal electrolyte Zn/Zn symmetric cells before cycling

	**Liquid**		**HCCE**	
	**Value**	**Error (%)**	**Value**	**Error (%)**
Before				
Rs	1.07	8.40	2.725	2.89
Rct	522.5	1.86	393.3	1.82
After				
Rs	2.722	3.66	2.683	2.88
Rct	1049	1.72	608.8	1.57

Table S4 Calculation of transference numbers from analysis of polarization experiments

**Cell**	**ΔV (mV)**	**R_O (Ω)**	**R_S (Ω)**	**I_O (μA)**	**I_S (μA)**	**t_+**
Liquid	*5*	521.5	1046.3	6.542	3.206	0.498
HCCE	*5*	390.6	606.1	19.61	10.32	0.644

Table S5 The fitting resistance results of symmetric cells for the liquid and the HCCE by the equivalent circuit at difference temperature

Symmetric Cells	Resistance(Ω)	0 ºC (Ω)	10 ºC (Ω)	20 ºC (Ω)	25 ºC (Ω)	40 ºC (Ω)
Liquid	*Rct*	8444	5039	1711	672.5	584.8
	Rs	20.57	11.92	9.766	6.116	6.098
HCCE	*Rct*	4857	2569	1435	1026	850.8
	Rs	6.374	5.255	5.305	4.617	4.94

Supplementary References

[S1] G. Fang, C. Zhu, M. Chen, J. Zhou, B. Tang et al., Suppressing manganese dissolution in potassium manganate with rich oxygen defects engaged high-energy-density and durable aqueous zinc-ion battery. Adv. Funct. Mater. **29**(15), 1808375 (2019). https://doi.org/10.1002/adfm.201808375

[S2] H. Li, Z. Liu, G. Liang, Y. Huang, Y. Huang et al., Waterproof and tailorable elastic rechargeable yarn zinc ion batteries by a cross-linked polyacrylamide electrolyte. ACS Nano **12**(4), 3140-3148 (2018). https://doi.org/10.1021/acsnano.7b09003

[S3] J. Evans, C. A. Vincent, P. G. Bruce, Electrochemical measurement of transference numbers in polymer electrolytes. Polymer **28**(13), 2324-2328 (1987). https://doi.org/10.1016/0032-3861(87)90394-6
